# A review of emerging physical transfection methods for CRISPR/Cas9-mediated gene editing

**DOI:** 10.7150/thno.43465

**Published:** 2020-04-15

**Authors:** Apresio K. Fajrial, Qing Qing He, Nurul I. Wirusanti, Jill E. Slansky, Xiaoyun Ding

**Affiliations:** 1 Paul M. Rady Department of Mechanical Engineering, University of Colorado Boulder, Boulder, CO, 80309, USA; 2 University Medical Center Groningen, University of Groningen, Groningen, The Netherland; 3 Department of Immunology and Microbiology, University of Colorado School of Medicine, Aurora, CO, 80045, USA

**Keywords:** physical transfection, CRISPR delivery, intracellular delivery, gene editing, transfection methods, micro/nanotechnology

## Abstract

Gene editing is a versatile technique in biomedicine that promotes fundamental research as well as clinical therapy. The development of Clustered Regularly Interspaced Short Palindromic Repeats (CRISPR) as a genome editing machinery has accelerated the application of gene editing. However, the delivery of CRISPR components often suffers when using conventional transfection methods, such as viral transduction and chemical vectors, due to limited packaging size and inefficiency toward certain cell types. In this review, we discuss physical transfection methods for CRISPR gene editing which can overcome these limitations. We outline different types of physical transfection methods, highlight novel techniques to deliver CRISPR components, and emphasize the role of micro and nanotechnology to improve transfection performance. We present our perspectives on the limitations of current technology and provide insights on the future developments of physical transfection methods.

## Introduction

Gene editing is a technique that manipulates gene function by modifying the sequence in the target organism's DNA. Current gene editing methods rely on targeting specific sites in an organism's genome using nucleases. There are three major engineered nuclease tools for gene editing: Zinc Finger Nucleases, Transcription Activator-Like Effector Nucleases, and Clustered Regularly Interspaced Short Palindromic Repeats (CRISPR)/CRISPR associated protein 9 (Cas9) 1-3. Variants of the aforementioned gene editing tools have also emerged such as CRISPR base editor, prime editor, Cas9 nickase and dead Cas9 4-7. To edit target genes, the engineered nucleases induce double-stranded breaks at specific sites in the genome. Since its introduction to mammalian cells, CRISPR has eclipsed other classes of engineered nucleases due to its efficiency and selectivity. Cas9 works in conjunction with a guide RNA (gRNA) to identify a target sequence, then cuts the particular DNA sequence. CRISPR/Cas9 also enables sequence deletion and insertion by providing a DNA repair template into the cell.

Delivery technologies for CRISPR/Cas9 are similar to transfection methods for nucleic acids. Transfection of CRISPR/Cas9 is usually conducted using either viral or chemical vectors 8,9. In addition, physical transfection allows direct delivery of CRISPR/Cas9. Physical transfection employs mechanical or electrical forces to create transient pores in the cell membrane, which enhances intracellular uptake of target molecules. Recently, novel physical methods for transfection have surged due to the development of microtechnology and nanotechnology 10-16. Nanostructure-mediated electroporation, for example, allows miniaturization of physical transfection to improve transfection efficiency and precision. It uniformly treats cells with minimal damage to cell viability in comparison to stochastic uneven cell permeabilization using bulk electroporation.

In this review, we thoroughly assess physical transfection methods for CRISPR delivery to mammalian cells. We provide a brief introduction of CRISPR as a gene editing method. We then highlight various physical transfection platforms for CRISPR with a special focus on recent advances using micro and nanotechnologies. Finally, we provide insights on challenges during physical transfection as well as the technology's future outlook.

## Gene-Editing Machinery: CRISPR

Originating from observations of the adaptive immunity of bacterial cells in their environments, CRISPR and CRISPR-associated proteins are currently at the forefront of gene-editing technology 17. Of the three types of CRISPR mechanisms identified, type II has the greatest application 18. In a bacterial cell, the type II mechanism stores DNA of previously encountered phages in its CRISPR locus between short palindromic repeats (~20 base pairs), which can be transcribed into CRISPR RNA that guides the Cas9 nuclease to cut and inactivate the invading bacteriophage 19. Synthetic versions of CRISPR RNA directs the Cas9 nuclease to produce site-specific DNA double-stranded breaks (DSBs) 19.

At the most basic level, CRISPR/Cas9 gene editing has two key components: the Cas9 nuclease, which creates the DSB, and guide RNA (gRNA) which “programs” Cas9 to cut specific sites. The DSBs created by specific nucleases can mainly be repaired by two different endogenous repair processes: non-homologous end joining (NHEJ) and homology directed repair (HDR) 20. The resulting gene editing product depends on the underlying repair process. NHEJ facilitates the repair of a double strand break by joining the nucleic acid fragments through enzymatic activity. NHEJ is a very error-prone process and frequently produces insertions or deletions (indels) at the repair junction 21. These indels can cause frameshift mutations, premature stop codons or nonsense mediated decay 22. HDR offers a more precise DSB repair by providing a DNA template containing the desired insertion or correction, and is flanked by homologous arms: DNA sequences matching those in the target insertion site 23. Therefore, HDR is utilized to construct knock-in gene edit or gene replacement. However, HDR efficiency is low relative to NHEJ. HDR relies on the presence of homologous DNA inside the nucleus, which only occurs in the S or G2 phase of the cell cycle 24. Details on the underlying mechanism of CRISPR for gene editing have been comprehensively reviewed elsewhere 25,26.

In most scenarios, CRISPR/Cas9 complexes are delivered to the cytoplasm of target cells. The CRISPR/Cas9 complexes need to cross both the cell membrane and the nuclear membrane in order for gene-editing to occur within the nucleus. A nuclear localization sequence (NLS) encoded in the plasmid vector or the Cas9 protein enables the CRISPR/Cas9 construct to enter the nucleus. Without an NLS, a construct can only enter the nucleus when the nuclear membrane breaks down during cell division 27.

CRISPR/Cas9 can be delivered in three formats: DNA, mRNA, and RNP. When choosing a format for transfection, many factors come into consideration, including efficiency and the length of expression needed. The duration of expression is largely affected by the speed of biological processes. Depending on the aims of the experiment, certain CRISPR formats may be preferred to others.

### DNA Format

DNA vectors require both transcription and translation before gene editing can occur. Typically, transcription rates in humans range from 6-70 nucleotides per second and translation rates are approximately 5 amino acids per second 28,29. DNA enters the nucleus and is transcribed into the gRNA and the mRNA encoding Cas9 protein. The Cas9 mRNA is translated into protein in the cytoplasm, binds the gRNA, and returns to the nucleus for gene editing. Cas9 protein is typically detectable within 5 hours following transfection, peaks after 24-48 hours, and sometimes takes weeks before expression returns to base levels 30. Due to the long half-life of Cas9 protein, the DNA format is preferred for experiments that require longer durations. However, the prolonged-expression of the Cas9 gene may also result in undesired off-target effects. Furthermore, because the Cas9 protein requires transcription in the nucleus, plasmid DNA may get incorporated into the genome. Plasmid DNA is larger than the mRNA or RNP formats discussed below, which can be problematic in certain transfection methods 23. In addition, plasmid DNA requires compatible promoters for gRNA and Cas9 expression. Although the use of other formats are becoming more frequent, plasmid DNA is yet to become obsolete due to its stability and expandability. Also commercial vectors often include fluorescent biomarkers that are useful for proof-of-concept experiments.

### mRNA Format

The mRNA format does not require transcription and is instead directly translated in the cytoplasm before gRNA and Cas9 localize to the nucleus. In comparison to plasmid DNA, mRNA is more time-efficient 31. The Cas9 nuclease is expressed one hour following transfection, and peak expression occurs in 5-7 hours 30. The mRNA format is preferred for experiments that require shorter duration, which also limits off-target effects.

### RNP Format

The ribonucleoprotein format is a pre-formed Cas9 protein and gRNA complex that does not require transcription or translation. This format is the most efficient and can function within a matter of seconds when paired with transfection methods such as electroporation. The RNP format has the lowest chance of having off-target effects due to its speed and efficiency and can also insert large DNA fragments. However, the components of the RNP may be costly to produce or purchase and they are prone to toxic contaminants 32.

## Transfection Methods

### Viral transduction

Virus-mediated transduction, as the term implies, makes use of viral vectors, typically a type of virus that is deemed safe to transfer plasmid DNA into host cells. Viruses that are commonly used include adenoviruses, adeno-associated viruses (AAV), retroviruses, and lentiviruses 33. **Table 3** shows some characteristics of the major viral vectors for gene delivery. *In vitro* application often relies on lentivirus to transfect cells due to its ability to penetrate the nuclear envelope without cell division. For *in vivo* gene delivery, AAV is preferable because it does not incorporate itself into the genome of the target cell and elicits milder immune response in comparison to other viral vectors 34,35. AAV is currently a dominant method for *in vivo* gene therapy and has gained the US Food and Drug Administration (FDA) approval to treat some rare genetic diseases 36,37. These viruses currently have a limited DNA packaging size which is around 10 to 18 kilobases (kb) in length 38-41. Meanwhile, the Cas9 protein itself contains 1,368 amino acids encoded by over 4.1 kb DNA sequences, and when combined with the sgRNA sequence, the total CRISPR system DNA is often too large for a single viral vector. 42. Therefore, the CRISPR DNA is usually delivered in multiple viral vectors adding extra time and cost to the transfection process.

### Chemical transfection

Chemical transfection uses chemical vectors such as lipid vesicles and polymer-based chemicals to introduce cargo into target cells 43. The chemical vector acts as a delivery vehicle that encapsulates genetic editing plasmid DNA and mRNA 44,45. Inorganic structures such as gold nanoparticles, carbon nanotubes, and graphene also show promising results in delivering nucleic acid 46-48. Chemical transfection is also able to deliver protein and other biomolecules. ThermoFisher Scientific has released a lipid-based chemical transfection reagent that is optimized to deliver Cas9 RNP complexes 49. Unlike viral transduction, the size of the cargo is usually not a concern when using chemical vectors. The lipid-covered cargo is introduced into the cell through endocytosis. The lipid vesicle facilitates endocytosis and protects the cargo from enzymatic degradation in the cytosol. When using lipofection, the cargo only reaches the cytoplasm, which is unfortunate for cargo such as plasmid DNA which needs to reach the nucleus to function. The chemical transfection is often ineffective for cells that are difficult to transfect such as immune cells and stem cells. The chemical method for these types of cells either fails to deliver the cargo or results in excessive cell death 50-52. Recently, vector-free chemical-based methods have also been reported. D'Astolfo *et al.* demonstrated the use of induced transduction by osmocytosis and propanebetaine (iTOP) for effective delivery of Cas9 RNP complexes into a wide variety of cells including mouse embryonic neural stem cells and dendritic cells 53. Soluporation technique utilizes solutions containing ethanol as a permeabilizing agent to deliver proteins and mRNA into mesenchymal stem cells and Jurkat cells 54. Other review articles have discussed this new class of transfection methods 55,56.

### Physical transfection

**Figure 1:**
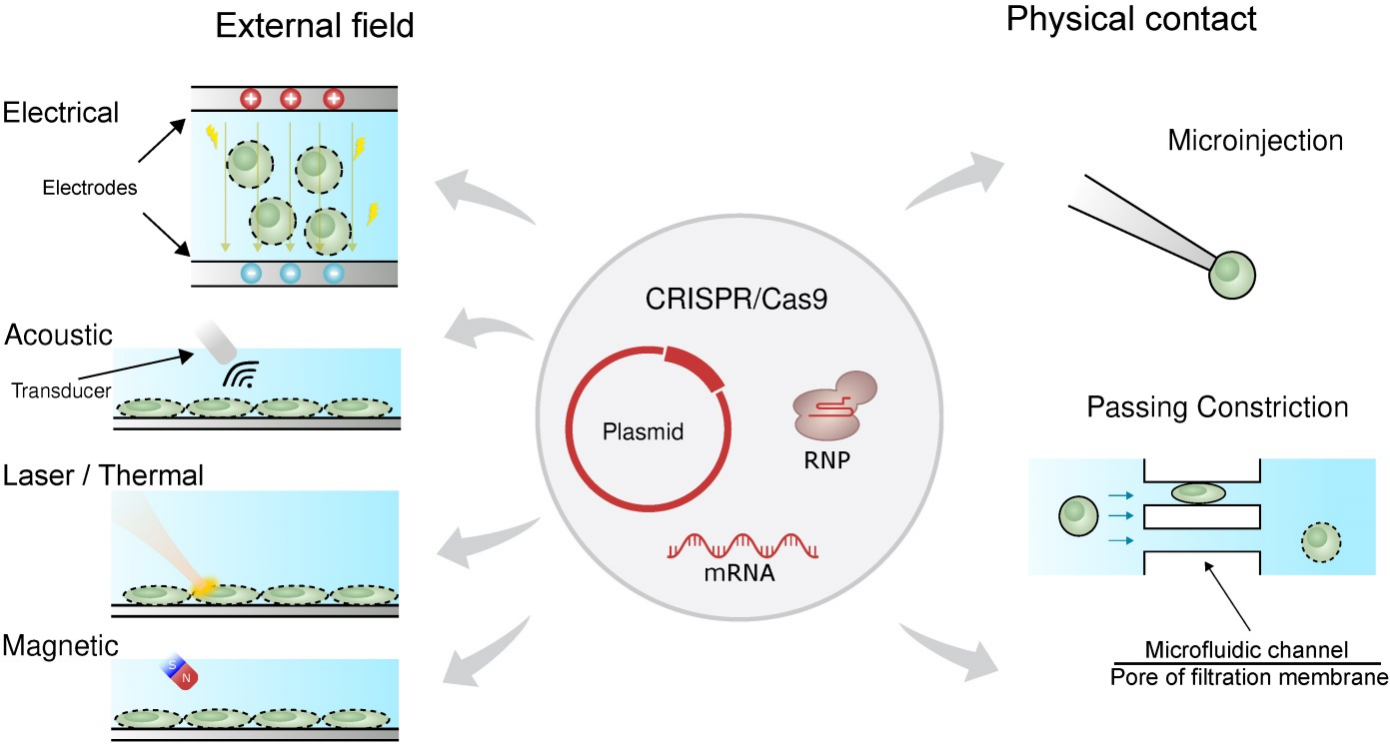
Physical forces responsible for CRISPR transfection. The CRISPR/Cas9 system can be delivered as plasmid DNA, mRNA or RNP. The driving forces for CRISPR delivery include external field such as electrical, acoustic, laser/thermal and magnetic forces. Direct physical contact such as microinjection and passing constriction can also mediate CRISPR delivery.

Physical transfection does not rely on the use of vectors 57-59. Consequently, unlike viral vectors, there is almost no limit to cargo size, and unlike chemical vectors, the rate-limiting step does not depend on cell endocytosis. **Figure 1** shows that physical transfection can harness energy from electrical, thermal, and mechanical forces. Applied forces compromise the cell membrane, allowing the cargo to diffuse into the cell and, in some cases, assist in active delivery of the cargo itself. Electroporation, for example, shocks the cell with an electric field that induces membrane perforation and drift forces to charged cargo such as plasmid DNA.

Despite the advantages of physical transfection, there are also limitations. *In vivo* physical transfection is still too invasive for human application, although special circumstances warrant further exploration 60. DNA vaccines, whose delivery technique is identical to gene editing, have been physically transfected *in vivo* into mice using gene gun injection and electroporation 61-65. DNA vaccine only requires intradermal or intramuscular delivery unlike many gene therapies for genetic diseases that are generally invasive 66. For gene editing, *in vivo* electroporation has demonstrated the delivery of genetic materials into retina and epidermis tissue in mice 67,68. Similarly, a silicon nanoneedle can deliver plasmid DNA encoding the vascular endothelial growth factor into the muscles of mice, promoting tissue neovascularization 69,70. However, due to its invasive nature, *in vivo* transfection usually involves delivery vectors.

Although CRISPR offers solutions to remedy genetic diseases such as muscular dystrophy and hemophilia 71,72, its *in vivo* administration raises some safety concerns. The effectiveness of CRISPR also comes with off-target effects that may cause unwanted mutations. Efforts are currently underway to ensure that the cargo reaches the target cells and to control the dosage of CRISPR *in vivo*. While the suitability of *in vivo* clinical application of gene editing may be hotly debated in the biomedical community, *in vitro* and *ex vivo* gene editing remain irreplaceable techniques in biomedical research.

Physical methods may also lend merit for *in vitro* delivery to hard-to-transfect-cells. A lot of important biomedical research require difficult to transfect primary cells such as neurons, stem cells, and immune cells. Editing these cells *in vitro* is an efficient approach to study unidentified human gene function 73.

In addition to *in vitro* genome editing, physical transfection is superior for *ex vivo* applications 74. *Ex vivo* genome editing is particularly beneficial for clinical efforts involving cell therapy. Cancer immunotherapy, for example, utilizes genome edited T-lymphocytes to recognize and attack tumor cells. Currently, there are several ongoing clinical trials using *ex vivo* engineered cells that rely on viral transfection for genetic modification. The projected cost is considerably high and requires a long time to generate enough cells for treatment. Recently, clinical trials using T cells engineered via physical transfection have demonstrated improved efficiency through high-throughput genome editing for cancer immunotherapy 75. Xu *et al.* reported a case study of *ex vivo* CRISPR-edited stem cell therapy via electroporation 76. Despite a small disruption of the target gene in the patient, the edited cells endure for more than 19 months and the patient did not develop further complications. Another study by Stadtmauer *et al.* reported a phase 1 clinical trial of cell therapy employing multiplex gene editing of T cells for three patients with refractory cancer 77. Using electroporation, they delivered the Cas9 RNP to disrupt endogenous T cell receptor (TCR) and programmed cell death protein (PD-1). The patient T cells were also transduced by lentiviral vectors to express cancer-specific TCR for tumor targeting. The engineered T cells remained stable in all patients for 9 months. One patient also showed a significant decrease in tumor size. **Table 4** summarizes recently completed and ongoing clinical trials which harness physical transfection to engineer target cells.

Considering the benefit of physical transfection for *in vitro* and *ex vivo* application, here we discuss novel physical transfection technology for genome editing that relies on membrane disruption and permeabilization. We emphasize the role of miniaturization and micro/nanotechnology for the emerging physical methods for genome editing 78. Furthermore, we assess the editing efficiency of the technology and its potential drawbacks.

### Mechanical transfection

Mechanical transfection, or mechanotransfection, uses mechanical forces such as physical contact with a solid structure or shear force from the surrounding fluid, to create pores in the cell membrane. The pores allow target materials to diffuse into the cytosol. In some techniques, such as microinjection, the buffer containing target materials are injected directly into the intracellular space.

Microinjection is a traditional example of mechanical transfection, wherein genes are injected into cells using a micrometer-sized capillary 79,80. Direct injection of genetic materials into the nucleus using microinjection enables rapid gene expression. Microinjection is practical for single-cell applications such as germline editing. Delivering CRISPR by microinjection has accelerated the creation of model organisms with desired phenotypes. Sheep with knocked-out muscle growth inhibition gene was created by injecting gRNA targeting the myostatin-encoding gene and Cas9 mRNA into the cytosol of sheep zygote 81. Microinjection of CRISPR has been efficacious in a wide variety of species including sand flies, zebrafish, mice, and pigs 82-85. Although microinjection is highly efficient, it is low throughput and an experienced technician is required to carefully perform the injections so the cells remain viable.

**Figure 2:**
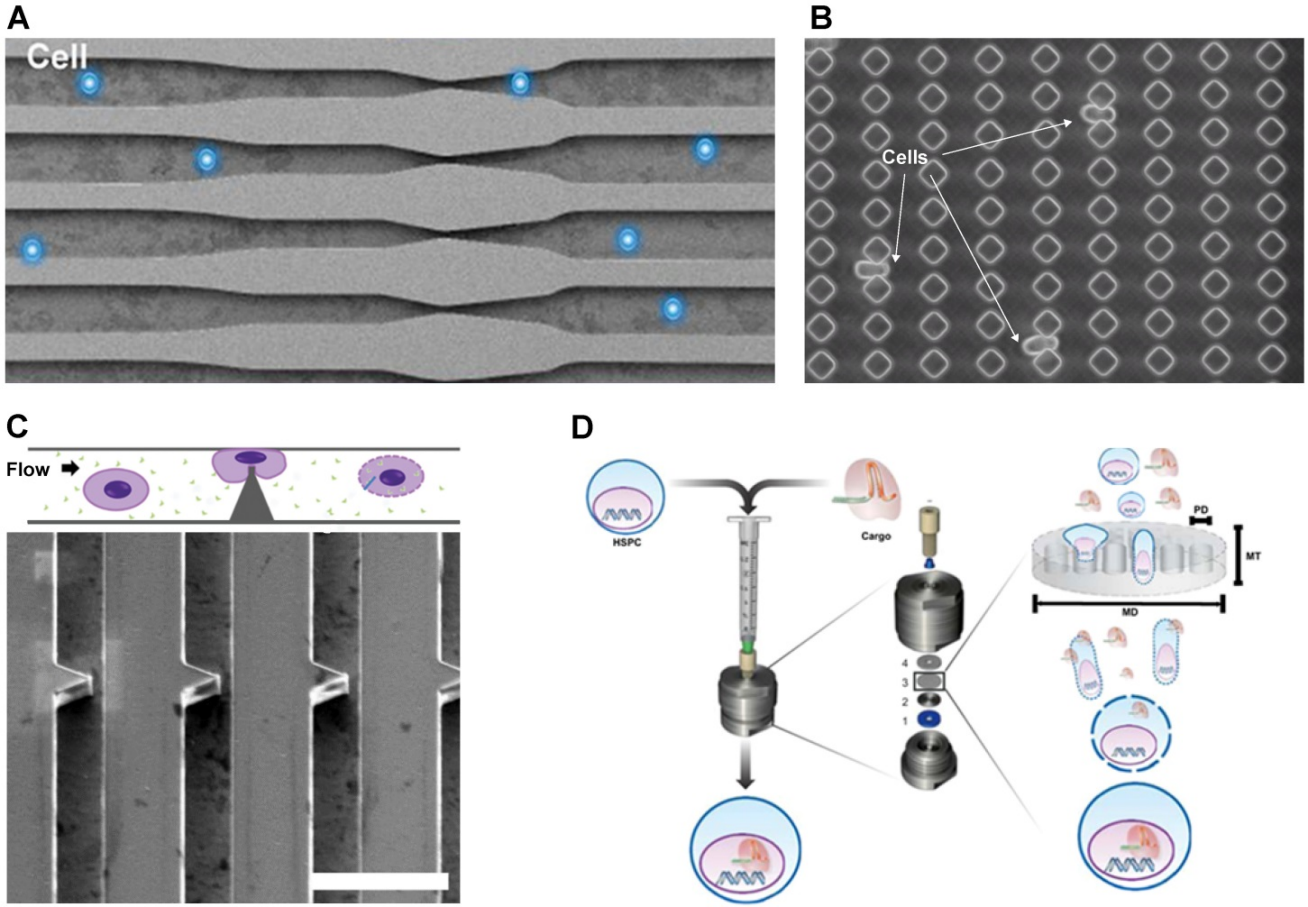
Mechanical transfection platform deforms cell membrane. **A.** Cell squeezing device. Reproduced with permission from Proceedings of the National Academy of Sciences USA 86 Copyright 2013. **B.** Workflow schematic and SEM image of sharp angle constriction made of silicon, scale bar: 30 µm. Reproduced with permission from Oxford University Press 87 Copyright 2017. **C.** Image of cells being deformed when passing through the constriction. Reproduced with permission from American Association for the Advancement of Science 88 CC BY-NC 4.0. **D.** Setup of TRansmembrane Internalization Assisted by Membrane Filtration (TRIAMF) method for CRISPR transfection. Reproduced with permission from Springer Nature 89 CC BY 4.0.

Micro and nanotechnology have been employed to develop a mechanical poration platform for gene-editing. Sharei *et al.* passed cells through a microfabricated silicon constriction to create temporary pores for intracellular delivery 86. The delivery materials and the cells were mixed together and flowed through the microfluidic channel as shown in **Figure 2A**. The constriction width is usually around half the cell diameter while the length varies from 5 to 30 µm. Qin's Group demonstrated the delivery of CRISPR into mammalian cells using a membrane deformation device made of diamond-shaped polydimethylsiloxane (PDMS) pillars with a constriction width of 4 µm (**Figure 2B**) 88. Inside the microfluidic device, cells passed through 10 identical constrictions. According to this study, a sharp angle of constriction preserved cell viability better than a curved constriction from the circle-shaped pillar. In addition, mechanical deformation effectively transfected plasmid DNA into cells that are traditionally difficult to transfect, such as lymphoma and stem cells. Passing cells through constrictions delivered an enhanced green fluorescent protein (EGFP) reporter plasmid to ~30% and 50% of SU-DHL-1 lymphoma cells and AB 2.2. mouse embryonic stem cells, respectively. The positive control, FuGENE HD lipid transfection reagent, delivered the EGFP plasmid to only ~5% of the SU-DHL-1 cells and 10% of the AB 2.2. cells. Moreover, the mechanically transfected AB 2.2. stem cells showed imperceptible change in Oct4 expression compared to the control group, suggesting that the method preserved the stemness of the cell. The cell membrane deformation method is clearly superior compared to the conventional chemical transfection.

Qin's group also managed to knockout EGFP through delivering CRISPR to the MDA-MB-231 and the SU-DHL-1 cells using the same microfluidic constriction platform 88. Delivery of the Cas9 plasmid and a single gRNA targeting EGFP resulted in knock out of EGFP in ~90% of the MDA-MB-231 cells and ~70% in SU-DHL-1 cells. However, the cells were passed through the device three times to increase the efficacy of the delivery, which is suspected to decrease the cell viability (<50%). In addition, the study did not assess the plasmid concentration in the buffer, the yield, cell loss, or the concentration of the cells.

Aside from plasmid DNA delivery, the membrane deformation technique can also deliver the RNP complex. A study by Han *et al.* assessed the effect of RNP concentration on editing efficiency 90. EGFP-expressing SK-BR-3 cells were mechanically transfected with RNP targeting the EGFP sequence. The data showed that increasing RNP concentration positively correlated with the editing efficiency, which reached maximum knockout efficiency at 2 µM RNP. To determine the applicability of the platform, RNPs targeting p38 mitogen-activated protein kinases were delivered into human breast cancer cells (MDA-MB-231 and SUM-159) and primary human T cells. The editing efficiency (defined as mutation frequency) for these cells were 43%, 47%, and 33%, respectively. The study also compared the editing performance between CRISPR encoding plasmid and RNP. Both formats achieved similar indel rates of ~40%. However, off-target mutation rates differed: plasmid transfection led to a mutation rate of 4.7% while RNP transfection only accounted for 0.8%.

Mechanical transfection has been successful for CRISPR delivery of hard-to-transfect cells, such as stem cells and immune cells, which are often employed in *ex vivo* cell therapy 87,89-91. **Figure 2C** portrays a 2 µm sharp-angle constriction device to transfect human hematopoietic stem cells (HSCs). The RNP construct was designed to target the C/EBPα gene, which contributes to acute myeloid leukemia 87. Although not explicitly quantitated, western blot assays revealed that 2 µM of the RNP greatly suppressed gene expression. Another method to transfect HSCs was to flow the cells through a membrane filter as shown in **Figure 2D** 89. Although this may vary with cell size, the most efficient membrane filter for intracellular delivery had a 7 µm thickness with an 8 µm pore diameter. Stem cells were transfected with CRISPR RNP to knockout β2-microglobulina marker in urine for blood disorders such as β-thalassemia and sickle cell disease. The optimal CRISPR RNP concentration was 25 µM, and up to 63% of the cells were edited with such treatment. For immune cell transfection, membrane deformation was applied to deliver a CRISPR RNP complex targeting the PD-1 gene, a negative regulator of T cell activity. In one study, 2 µM of RNP complexes were delivered to T cells, achieving 35% PD-1 surface expression knockout and 36% cells with surveyor mutations 90. Similar results of PD-1 knockout were demonstrated with a cell squeezing platform 91. A maximum editing efficiency of 55% was achieved when using 100 µg/mL (~0.625 µM) RNP complexes with 2 gRNAs against PD-1 in human T cells.

### Electroporation

Shocking mammalian cells with an electric field, known as electroporation, was developed to effectively transfer nucleic acids into cells 96. The process of applying an electric field to transfer genes into a cell is complicated 97. Direct observation showed that elevated voltage creates instability and pores in the cell membrane through which small molecules diffuse 98. For plasmid DNA electroporation, the DNA aggregates with the cell membrane and activates endocytosis 99. The type of molecule being introduced, the amount of voltage applied, the length of the electrical pulse, the buffer solution, and the type of cells used are important parameters that yield reversible permeabilization of the cell membrane and effective cargo delivery.

Along with lipofection, electroporation is currently one of the most commercially available gene transfection methods, due to its simplicity and efficacy. Oftentimes, researchers opt for electroporation because it is more effective for cells such as immune cells and stem cells. However, some electroporated cells may lose viability due to excessive heat, pH changes, and ionic imbalances. The cargo delivered can also affect cell health. Large plasmids decrease cell viability compared to small plasmids 100. In addition, the buffer solution used during transfection affects cell viability. Standard buffers such as Phosphate Buffered Saline (PBS) or Hank's Balanced Salt Solution for electroporation can cause significant cell death 101. In response to this issue, manufacturers have developed expensive optimized electroporation buffers. One optimized electroporation buffer preserves cell viability despite inherently causing some cell death 89. Lengthy exposure to another buffer reduces transfection efficiency 102. Exposing electroporated T cells to BioTechnology eXperimental Research buffer (BTX), for 15 minutes reduced efficiency of mRNA transfection by 20%. One hour of exposure to BTX buffer cut transfection efficiency to 70%. Regardless of its many issues, electroporation is useful for transfecting cells that are generally difficult to transfect.

Electroporation has delivered the CRISPR system into a wide array of cell types with varying degrees of success 103,104. Bulk electroporation can deliver the CRISPR system in the form of a plasmid, mRNA, and RNP for both knock-in and knock-out of targeted sequences 105-108. Multiple sequence knock-out has also been demonstrated by delivering more than two gRNAs with the Cas9 protein 109. The CRISPR RNP complex is more frequently electroporated relative to plasmid or mRNA, likely because it is small, easy to deliver, and the gene editing occurs faster. Optimization of CRISPR RNP using conventional electroporation can disrupt 98% of target genes in primary T cells 110.

**Figure 3:**
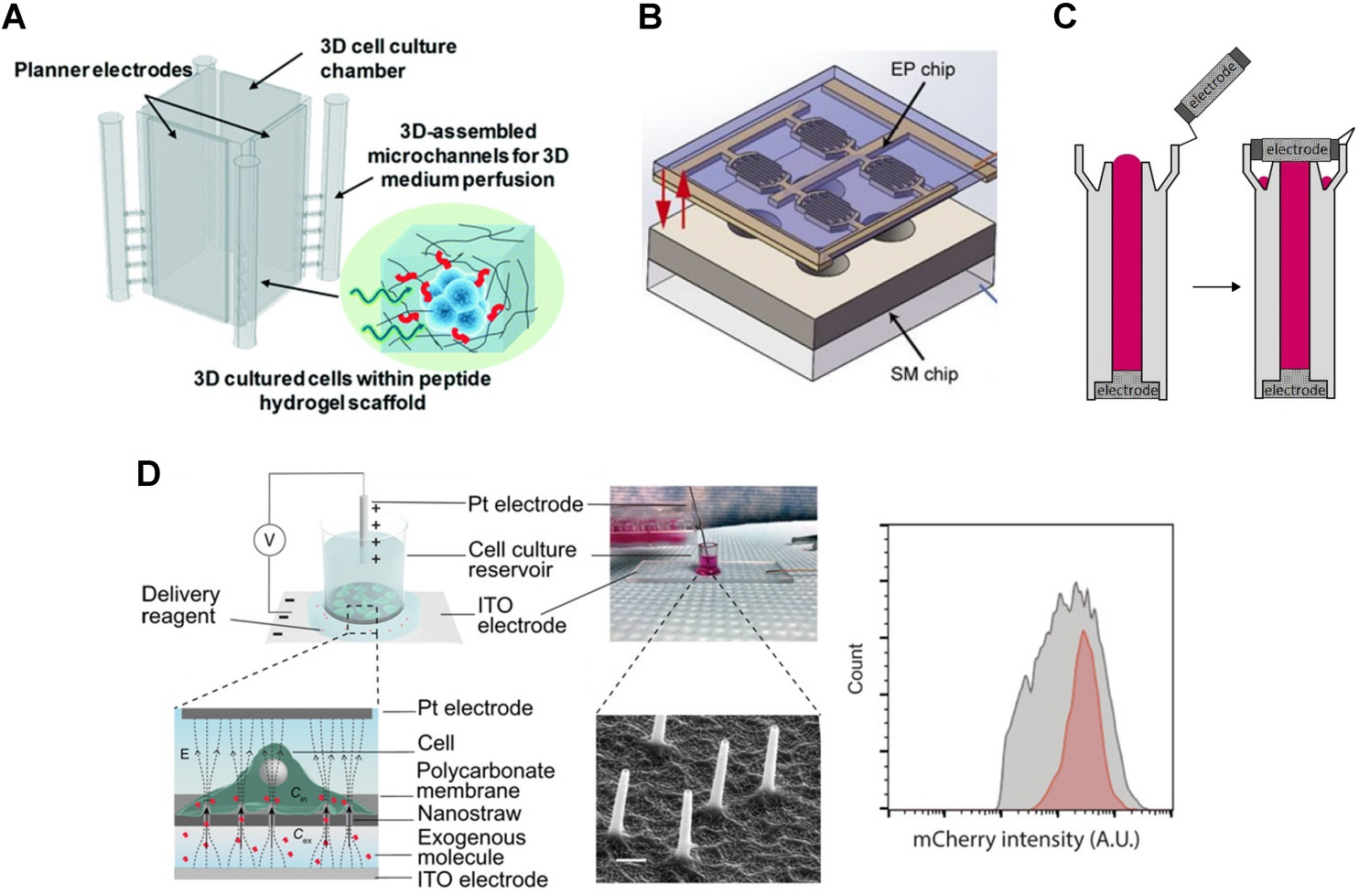
Novel electroporation platform for transfection. **A.** Schematic of 3D microfluidic electroporation system. Reproduced from Ref. 92 with permission from The Royal Society of Chemistry. **B.** Design for *in situ* electroporation microsystem. Reproduced with permission from Springer Nature 93 CC BY 4.0. **C.** Cuvette design for tube electroporation. Reproduced with permission from Springer Nature 94 CC BY 4.0. **D.** Nanostructure electroporation system (left) and corresponding mRNA transfection efficiency showing higher uniformity (red) compared to lipofection (gray) (right). Reproduced with permission from American Association for the Advancement of Science 95 CC BY-NC 4.0.

New electroporation tools enable creative applications of CRISPR transfection. The 3D μ-electrotransfection system merges 3D printing with microfluidics and is used to transfect *in situ* HEK 293 cells cultured in a 3D microenvironment (**Figure 3A**) 92. The scaffold for culturing cells consists of peptide hydrogel matrix. This limits transfection efficiency due to the large size of the plasmid and limited molecular transport in porous hydrogel despite the use of a high CRISPR/Cas9 plasmid concentration (200 μg/mL). Similar *in situ* CRISPR transfection using electroporation is demonstrated in multi-well 2D cell culture systems (see **Figure 3B**), although transfection efficiency is not quantified 93.

Conventional electroporation often causes non-uniform electric field distribution and bubbles inside the cuvette. To address this challenge, Xu *et al.* modified the cuvette by incorporating electrodes to ensure no liquid/air interface inside the tube-shaped cuvette, **Figure 3C** 94. Using this tube electroporation, together with 0.5 μM Cas9 RNP and 0.85 μM gRNA, up to 90% of a point mutation was introduced in human mesenchymal stem cells, human induced pluripotent cells, and human primary T cells.

Miniaturization and nanotechnology offer practical solutions to address the disadvantages of conventional electroporation. Incorporating nanostructures, such as nanochannels or nanostraws, enhanced the local electric field, thus lowered the operating voltage and prevented bubbles formation 95,111,112. Spatial and dosage controls are also tuned by the type of electrode used and the voltage applied. Using a nanostraw electroporation platform (**Figure 3D**), Cao *et al.* demonstrated 85% mRNA transfection efficiency and more than 90% cell viability 95. Compared to lipofection, the device transfected mRNA more uniformly as shown in **Figure 3D**. The nanoelectroporation device introduced gRNA against the human housekeeping PPIB gene and 10 µM of GFP-tagged Cas9 protein into HEK 293 cells. 90% of the cells internalized the RNP complexes as determined by the GFP signal. Meanwhile, the editing efficiency was 31% and 33% as determined by T7E1 assay and Sanger sequencing, respectively.

In the nanoelectroporation platform, a tight-knit interface between the cells and the micro/nanostructures is necessary for the local field enhancement to take effect. Spatial control of the cell using an optical tweezer or the dielectrophoretic effect assists in ensuring this requirement 112,113. Aside from physically controlling the cell position, nanostructures can also be coated with polymers or proteins known to improve cell adhesion 101. Increasing the concentration of these adhesion molecules increases the strength of cell adhesion. However, if the concentration is too high, cell viability will decrease when the voltage is applied due to adhesion forces between the cell and the surface. Therefore, the concentration of the adhesion molecules must be optimized to create just enough attraction between the surface and the cell membrane without leading to excessive cell injury.

In order to create micro and nanostructures, researchers have relied on a costly lithography-based method. To tackle this issue, Cao *et al.* developed a commercially available and affordable nanopore electroporation device made of a water filtration membrane 101. The use of this device resulted in gene editing of about 25% of HeLa and Jurkat cells with 10 µM RNP against the housekeeping gene PPIB. The cell viability after transfection in cell culture medium was around 95%. By comparison, bulk electroporation using PBS or cell culture medium as a buffer lead to less than 55% cell viability. Meanwhile, HeLa's cell viability dropped to 50% using lipofection.

### Acoustoporation

**Figure 4:**
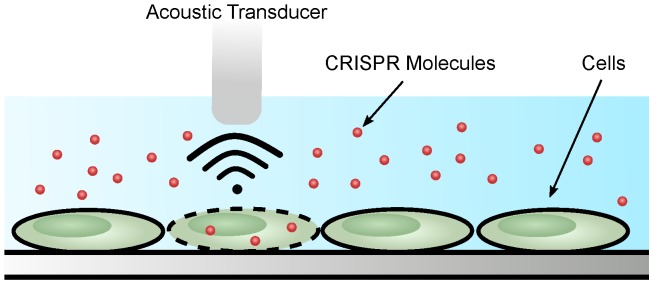
Acoustoporation methods for cell transfection.

Acoustoporation or sonoporation is a method that causes transient permeabilization of the cell membrane using sound (acoustic) waves 114 (**Figure 4**). The mechanism by which acoustic waves obstruct the cell membrane is similar to cavitation bubble formation; specifically, by inducing shock waves and streaming of the surrounding liquid medium 115,116. Oftentimes, microbubbles introduced into the medium increase membrane perforation efficiency. Under the acoustic field, the bubbles inflate or shrink depending on the pressure. The bubbles burst at high pressure causing shock waves. The combination of microbubbles and sonoporation leads to lower acoustic pressure needed for efficient transfection 117.

Using the supportive role of microbubbles, ultrasound waves transfected plasmid encoding Cas9 and gRNA to knockout c-erbB2 in human endometrial cancer (HEC)-1A cells 118. The microbubble suspension was mixed with 10 µg of plasmid and added to the cell medium. Upon transfection, the C-erbB-2 mRNA expression dropped to 57%.

In the absence of microbubbles, acoustic waves create pores by causing strong streaming in the extracellular environment. Surface acoustic waves (SAW) of 10 MHz were applied to cells to enhance molecular uptake 119. Unlike SAW, bulk acoustic waves at that frequency generate cavitation. Using SAW, HeLa cells had a transfection efficiency of 40% for siRNA-liposome complex. Meanwhile, without SAW exposure, mixing cells with siRNA-liposome complex only achieved 20% internalization. In yet another study, a much higher frequency at the hypersound level induced cell membrane perforation. A nanoelectromechanical resonator was developed to generate an acoustic wave at 1.6 GHz, creating membrane pores of around 200 nm 120.

CRISPR plasmids delivery via sonoporation has been demonstrated via high-frequency bulk acoustic waves (150 MHz frequency) applied to HeLa and HEK 293 cells 121. This acoustotransfection system introduced a pCas9 plasmid with delivery efficiency up to 40%. Transfection efficiency was contingent upon plasmid size and plasmid concentration. The high frequency ultrasound transducer could also knock-in target genes, although editing efficiency was not quantified in this study.

### Magnetotransfection

Magnetic field-induced intracellular delivery, called magnetotransfection or magnetofection, can occur with or without assistance of magnetic particles 122. High magnetic field pulses permeabilize cell membranes mainly due to electromagnetic heating 123-125. Generating a strong magnetic field also induces an electric field on the body of the target sample. The mechanism of this contactless strong magnetic field on cell permeabilization is still poorly understood. Currently the permeabilization efficiency of the high magnetic field pulse is low.

Conversely, utilizing magnetic nanoparticles conjugated with target molecules intensifies the intracellular delivery into target cells when exposed to a magnetic field 122. Commercial magnetofection kits contain spherical magnetic nanoparticles with a positive charge to ease complex formation with nucleic acids. Recently, a sharp magnetic particle, termed a nanospear, was developed to deliver plasmids with high spatial resolution 126. An external magnetic field guides the nanospear precisely into a single-cell target.

CRISPR delivery has been demonstrated using magnetic nanoparticles. Plasmids encoding Cas9 and gRNA targeting the H11 locus of porcine fibroblasts were combined with polyethyleneimine-coated magnetic particles 127. Magnetofection was applied to porcine fetal fibroblasts at different plasmid concentrations. The editing efficiencies of the magnetofection, quantified as indel frequency, were 15% and 27% for plasmid concentration of 375 ng/mL and 750 ng/mL, respectively. While increasing plasmid concentration corresponded to a higher transfection rate, increased plasmid internalization considerably decreased cell viability to 40%. A similar trend of cell viability reduction upon high plasmid concentration also occurred using lipofection.

The magnetic force is also beneficial for on-demand control of magnetofection. A proof-of-principle study in an *in vitro* model showed that stimulating magnetic nanoparticles with a magnetic field could facilitate particle migration across the blood-brain barrier 128. After passing the blood-brain barrier, a CRISPR plasmid was released by an alternate magnetic field trigger. *In vivo* control of the magnetofection was demonstrated by combining baculoviral vectors with the magnetic nanoparticles 129. The baculoviral vector encoded the Cas9 and gRNA to knock down the Vegfr2 gene, which is responsible for cell proliferation. Initial tests were conducted using magnetofection *in vitro* to edit Vegfr2 in Hepa 1-6 cell, a murine hepatoma cell line. The results showed that the magnetic particles induced the rapid intracellular uptake, thus preventing baculoviral vector inactivation by immune system. This technique was capable to cut the Vegfr2 gene with an efficiency up to 30%. Following *in vitro* test, *in vivo* evaluation was conducted by intravenously injecting the particle-DNA complex. The magnetic field stimulation near the tumor site encouraged magnetic nanoparticles to aggregate, thus increasing the internalization of the plasmid by tumor cells. After transfection, gene-edited cells containing EGFP reporter were harvested. These cells were successfully edited at 30% efficiency. Moreover, this system successfully edited target tumor cells with a maximum indel rate of 4.7%.

### Laser optoporation

A laser focused on the cell membrane can generate transient pores that allow extracellular molecules into the cytosol. Laser optoporation was first successfully performed in 1984 to transfect normal rat kidney cells 132. Pore size is influenced by laser spot size, laser power, and pulse duration. Laser poration is highly efficient at the single-cell level.

**Figure 5:**
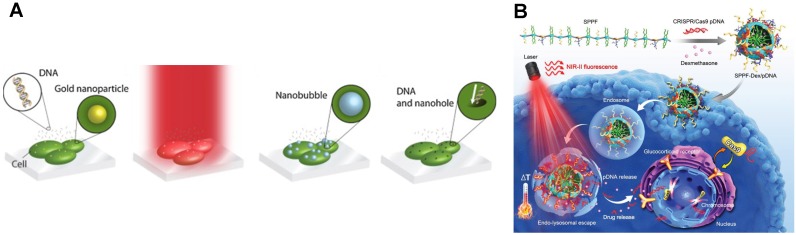
Laser optoporation methods for cell transfection. **A.** Workflow of transfection principles using laser optoporation. Reprinted from 130, Copyright 2013, with permission from Elsevier. **B.** On demand transfection triggered via laser and responsive particle. Reproduced with permission from 131 Copyright 2019 Wiley.

Irradiating metallic nanostructures using a laser triggers plasmonic effects that can porate cell membrane. **Figure 5A** shows the step-by-step mechanism of laser-plasmonic transfection. The plasmonic nanostructure absorbs energy from laser irradiation and converts it into an explosive bubble due to the rapid localized heating 133. The resulting shock wave from the bubble collapse leads to cell membrane poration, allowing biomolecules to enter the cells 134. Gold nanoparticles are the most common type of thermoplasmonic particle to mediate laser plasmonic poration 130. They can deliver target molecules, such as nucleic acids, into cells. While gold nanoparticles are not inherently toxic for the cell, it can be internalized and remain within the cell, which eventually lead to an unexpected response 135.

To prevent nanoparticle internalization, the thermoplasmonic structures are made out of a steady substrate, such as a gold nanoparticle layer or metallic nanostructures 136-139. For efficient perforation, a strong contact between the cells and the substrate is required. Therefore, prior to transfection, the adherent cells are cultured overnight on the substrate. Meanwhile, suspension cells do not anchor to the substrate effectively, and thus require alternative methods. Similar to the nanoelectroporation platform, the suspension cells are centrifuged onto the substrate to achieve a sufficient cell-substrate contact. Aside from centrifugation, sharp-tip metallic nanostructures are integrated with microwells for self-alignment of the cells with the tip position 140. This platform delivers lipid vesicles containing EGFP plasmids to Ramos B cells with a transfection efficiency of 58%, while using lipid vesicles alone only has a transfection efficiency of 24%.

Laser-based plasmonic poration technique facilitates CRISPR transfection into mammalian cells. gRNA targeting mouse CCR7 was delivered to SC1 cells expressing Cas9 and mouse CCR7 gene by perforating the cell membrane using laser-irradiated 200 nm gold nanoparticles 141. Similar to other physical transfection methods, the editing efficiency is influenced by gRNA concentration in the buffer solution. Maximum editing efficiency, assessed by flow cytometry seven days after treatment, was 30% at a 5 µM gRNA concentration. Transfections of gRNA and RNP complexes were also demonstrated using this technique. SC1 cells expressing mouse CCR7 were transfected with RNPs targeting the mouse CCR7 gene. Interestingly, the sgRNA-RNP complex concentration was not strongly correlated with the resulting editing efficiency, which reached a maximum of 22% with RNP complex concentration of 2.5 µM. Increasing the RNP complex concentration to 5 µM resulted in a lowered editing efficiency of 15%. The study tested the efficacy of the technique to deliver CRISPR to primary mouse CD8^+^ T cells. 5 µM of RNP complexes targeting mouse chemokine receptor CXCR3 were delivered to mouse CD8^+^ T cells, achieving a knock-out efficiency of 4%.

Lasers are also useful for on-demand delivery of the CRISPR system as illustrated in **Figure 5B** 142. Plasmids encoding Cas9 and gRNA were conjugated with gold nanoparticles and then the plasmid-particle complexes were encapsulated inside a lipid vesicle 143. This formulation knocked out the Plk-1 gene in A375 cells. Upon laser irradiation, there was a 65% reduction in Plk-1 expression in A375 cells exposed to the lipid vesicle/gold nanoparticle/plasmid hybrid. A similar CRISPR delivery system was constructed using a semiconducting polymer 131. Instead of visible light laser, the semiconducting polymer absorbs energy from nearby infrared laser irradiation triggering the release of cargo materials. *In vitro* tests of this technique to deliver Cas9 plasmid and GFP gRNA into HCT116 cells disrupts 52.1% of the GFP expression in laser-irradiated cells.

## Application of Genome Editing via Physical Transfection

### *In vitro* study of gene function, model organism generation, and tissue engineering

Gene editing can correct disease-inducing mutations in model organisms. For example, a mutation on the Crygc gene caused cataract in mice 144. The Crygc mutation was corrected by microinjection of CRISPR targeting the mutant allele in the mouse zygote. Aside from microinjection, CRISPR RNP electroporation of zygotes (CRISPR-EZ) also offers a cost-effective way to generate model organisms 145-147. As a proof-of-concept study, the Tyr gene was edited to generate albino mice offspring. CRISPR EZ outperformed microinjection in terms of the number of edited offspring and the number of mice born. Moreover, the study argued that the use of electroporation methods to generate edited embryos required less training for the user.

The physical transfection of CRISPR accelerates disease modelling studies via tissue engineering. CRISPR plasmids and homologous recombinant donor plasmids were delivered via electroporation to human intestinal organoids 148,149. The study found that chromosomal instabilities of the cells in the organoid were required to induce the invasive behavior of cancer. Moreover, due to improved efficacy in difficult-to-transfect cells, physical transfection was employed to facilitate CRISPR delivery to stem cells for tissue engineering. A CRISPR plasmid was electroporated into human induced pluripotent stem cells (iPSC) to knockout the HIF-1A gene, a transcription factor which controls oxygen homeostasis 150. After transfection, the iPSCs were differentiated into endothelial cells. 3D model tissues were then fabricated using the endothelial cells and hydrogel. The successfully formed vascular tissue responded to ischemic conditions by decreasing its lumen size diameter. In depth discussion of CRISPR gene editing for stem cell applications has been discussed elsewhere 151.

### *Ex vivo* cell therapy

Introduction of recombinant genes into T cells *ex vivo* is the initial step in some cancer immunotherapies. Successful tumor ablation has been shown by creating T cells with a chimeric antigen receptor (CAR) that recognizes CD19 and CD20 proteins on malignant B cell lymphomas 152. To engineer CAR T cells, viral vectors are usually employed to introduce the antigen receptor gene. Despite its efficacy, the production line of engineered T cells using viral vectors is costly. Production cost estimates of autologous T cell therapy are around 400,000 USD per treatment 153. In addition, despite the effort to develop viral vectors that package more DNA, they currently have limited DNA packaging capacity 41.

As described above, cargo is most efficiently introduced by physical transfection into hard-to-transfect cells. Studies have shown that bulk electroporation transfects CRISPR effectively to human primary immune cells. However, recent findings suggest that bulk electroporation is toxic to T cell function 91,154. While electroporation has been optimized for various cell types since its invention in the 1970s, the technology's harmful side effects on cells can be problematic.

In contrast, using mechanical transfection to deliver CRISPR in T cells helps preserve their functionality in tumor treatment 91. Microfluidic squeezing delivered CRISPR RNP complexes targeting the PD-1 gene in human T cells. A head-to-head comparison between electroporation and cell squeezing showed that both techniques edited the gene at a similar efficiency of 50%. However, electroporation caused more excessive misregulation of genes than mechanical transfection. A false discovery rate (FDR) and q-value analysis unveiled that 34% of the assessed genes were mis-expressed after electroporation, in comparison to 9% after mechanical transfection. Further investigation using a murine tumor model revealed that PD-1 knockout T cells edited via mechanical transfection controlled tumor growth, unlike electrotransfected cells despite similar editing efficiencies. This study emphasized the role of transfection methods in the functionality of *ex vivo* edited cells, implying the importance of evaluating cell phenotype upon intracellular delivery.

## Challenges and Outlook

Any successful transfection method poses distinctive concerns and limitations. Virus-mediated transfection has a limited cargo size, possibly induces immunogenicity, and is relatively expensive. On the other hand, chemical transfection is more flexible in regards to cargo size, but is often ineffective for difficult-to-transfect cells. Meanwhile, physical transfection offers a delivery method wherein efficiency is neither strongly dependent on cargo size nor cell type.

The performance of classic physical transfection methods such as bulk electroporation is impaired mainly due to its exorbitant perturbation to the cells treated. Currently, conventional bulk electroporation is arguably the most widely used physical transfection method. This is evidenced by the fact that manufacturers have optimized numerous parameters to ensure the highest delivery efficiency, including voltage, pulse length, amount of plasmid/cargo, and type of buffer. For non-clinical applications, the major goal of transfection is to establish edited genes in the target cells with a considerable amount of healthy cells left upon treatment. This purpose is effortlessly achieved using electroporation. However, for therapeutic application, there are some cell functions that must be preserved in treated cells. Unfortunately, physical transfection that was deemed effective to preserve cell viability may cause a significant untargeted change in gene expression. For example, electroporation of human T cells induces gene alteration that decreases the immune activity toward malignant tumors *in vivo* 91. During electroporation, cells are briskly exposed to a high electric field that permeabilizes the cell membrane. Under such condition, intracellular compartments and all the organelles also experience the electric field which may damage their integrity. The electric field also causes rapid molecular transport that destabilizes the ionic composition of the cells. Treating the cells with an electroporation buffer improves cell viability compared to standard cell culture buffers such as phosphate buffer saline, or Opti-MEM 155,156. Unfortunately, the exposure of cells to the electroporation buffer may undermine transfection efficiency. Prolonged exposure of human primary T cells to BTX electroporation buffer leads to more than a 70% decrease in transfection efficiency of mRNA 102. Similar findings have shown that HSCs treated with an electroporation buffer without the presence of delivery cargo has only 50% recovery rate, indicating a slower proliferation and metabolism 89. Fundamental studies on the impact of electroporation and electroporation buffers to cell functionality, beyond cell viability, are urgently needed in order to translate the technology to clinical application.

Micro and nanotechnology has spurred the development of superior physical transfection methods. Nanoscale electroporation mediated by nanostructures allows for a localized electric field, which ensures minimal perturbation on the cell functionality 157. Highly efficient mechanical transfection is also possible due to the advent of microscale engineering. Microfabricated fluidic channels with designed constriction drive transient permeabilization, thus allowing the uptake of extracellular molecules. The transport of molecules during mechanical transfection relies on diffusion, which is dependent on the molecule concentration and size. Diffusion-mediated transport is particularly slow for large molecules, such as DNA plasmids. To increase delivery efficiency, the concentration of the plasmid must be increased to the saturation point. Unfortunately, this approach is not cost effective, and high plasmid concentration in the buffer may induce unwanted toxicity. To overcome this problem, the plasmid can form complex with polymers or lipid vesicles to reduce the overall size of the cargo. Packing the plasmid inside a lipid vesicle or conjugating it with cationic polymers can also aid in protecting plasmid integrity upon cytosolic uptake.

Editing efficiency is a crucial parameter to determine the success of gene editing. It results from a complex interplay between the transfection methods as well as the designed CRISPR machinery. High transfection rates are required for high gene-editing efficiency, although they do not guarantee it. Bošnjak *et al.* demonstrated that despite the successful transfection of CRISPR RNPs, only 65% of treated cells showed genetic modification 141. Cao *et al.* reported similar findings 95. Even with a high rate of Cas9 delivery at 90%, editing efficiency was only 33% in HEK 293 cells.

One factor that potentially contributes to ineffective editing may be the guide RNA design 94,158. Therefore, to achieve high editing efficiency, multiple gRNA need to be designed and tested. Delivery of multiple gRNA sequences targeting the same site may also increase gene-editing performance. In addition to gRNA design, dosage of administered CRISPR formats also affects the efficiency of editing in the treated cells. Increasing the concentration of the plasmid, or using the mRNA or RNP formats as opposed to the DNA format usually improves the editing efficiency. For these reasons, in addition to creating a more effective transfection platform, optimization of the CRISPR formulation is also needed to achieve better precision in gene editing.

The efficiency of transfection and the throughput of processed cells are areas that should be considered for improvement in future technology. Indeed, high-throughput cell engineering is needed for clinical applications, such as *ex vivo* T cell immunotherapy. Due to limited initial quantities of cells for this application, the transfection process should aim to limit cell death and preserve function as much as possible. Upon transfection, *ex vivo* processed cells may need to undergo expansion to achieve a certain initial cell population prior to intravenous administration into the patients. This cell expansion process is inefficient and can contribute up to 75% of the time spent processing the engineered cells 159. Currently, there are several commercial devices for physical transfection in various stage of development (see **Table 5**). To increase the throughput of conventional electroporation device, companies develop an automated version or even a flow-through electroporation. Furthermore, companies are developing products based on other physical transfection methods. SQZ Biotech is developing a cell therapy product that consists of antigen presenting cells for immunotherapy. The company uses a microfluidic constriction device to deliver protein into PBMCs. Currently, many other companies commercializing physical transfection technologies are still in the development and startup stages.

Multiple physical approaches can be combined to increase the transfection efficiency without compromising cell health. Hybrid approaches integrate different physical forces to synergistically improve the overall transfection performance, while also minimizing the limitations of each physical approach. Ding *et al.* demonstrated that combining membrane deformation and electroporation can deliver plasmid DNA with rapid gene expression 160. This approach is minimally invasive due to the low voltage applied in comparison to bulk electroporation. The presence of an electric field, upon membrane disruption, assists gentle poration of the nuclear envelope, and the subsequent molecular transport of the plasmid to the nucleus. Similarly, Meacham *et al.* implemented acoustic shear poration in conjunction with electrophoretically driven plasmid DNA transports 161. The active transport of plasmid DNA significantly improved transfection performance with no reduction of cell viability.

In the future, *in vivo* transfection can be improved by combining stimuli-responsive materials with the physical approach. Zhu* et al.* demonstrated these using magnetic nanoparticles combined with baculovirus complexes to spatially control CRISPR transfection in mouse models 129. Smart materials that respond to external fields will also benefit the development of *in vivo* CRISPR delivery with spatiotemporal activation. *In vitro* demonstration of such systems utilize ultrasound-propelled nanomotors 162, an active material that only requires 0.6 nM concentration of RNP complexes to knockout the target gene.

In summary, physical transfection offers unprecedented advantages for CRISPR delivery. We have thoroughly discussed various physical transfection methods and their gene-editing performance using the CRISPR method. **Table 7** shows that physical transfection can achieve editing with an efficiency exceeding 50% with minimal damage to cell viability. We have established that the utilization of micro and nanoscale technology has incentivized the development of improved physical transfection platforms by increasing delivery efficiency while minimizing cell perturbation. This technology is especially valuable for *in vitro* and *ex vivo* cell engineering. In the future, physical transfection may assist in clinical application via *ex vivo* cell engineering production as well as *in vivo* targeting in conjunction with responsive carriers.

## Figures and Tables

**Table 1:** Glossary

Adherent cell	Also termed anchorage-dependent cell, is a type of cell which grows and spreads by adhering to the bottom surface of a tissue culture flask. Tissue-derived cells are commonly considered as adherent cells.
**Bulk acoustic wave**	A type of acoustic wave propagating inside the materials medium. Conventional sonoporation uses bulk acoustic wave for transfection.
**Dendritic cell**	A type of immune cell whose main function is to process and present antigen to activate adaptive immune response. Viral transfection to dendritic cell (DC) increases DC's immunogenicity and maturation.
**Dielectrophoresis**	A movement of neutral particle in a medium with different polarizabilities under the influence of a non-uniform electric field.
**Endocytosis**	An active internalization process of substances from the cell's environment into the cell by forming a membrane-bounded vesicle.
***Ex vivo***	Procedure in which cells or tissues are taken out from a living organism to receive treatment and subsequently returned to the living body.
***In vitro***	Procedure in which cell or tissues are isolated and treated outside of their natural biological environment. It may involve growing cells in test tube, flask, or petri dish to facilitate a more convenient analysis.
***In vivo***	Procedure that is conducted in a whole living organism as opposed to parts of organism. *In vivo* study allows observation of overall effects of a particular treatment.
**Lymphocytes**	A type of white blood cell found in the lymphatic system. There are two types of lymphocytes: B lymphocytes which create antibodies and T lymphocytes which attack infected and tumor cells.
**Microtechnology**	Technology at the micrometers (10^-6^ meters). Representation of microtechnology in transfection method includes the use of microfluidics and microelectrodes.
**Nanotechnology**	Technology at the nanometers (10^-9^ meters). Representation of nanotechnology in transfection method includes the use of nanoparticles, nanoneedles, nanostraws, and nanotubes.
**Natural killer (NK) cells**	A part of innate immune system that can kill infected and tumor cells.
**Nuclease**	In gene editing, nuclease acts as an enzyme that cleaves the bonds between nucleotides in nucleic acids and allows the editing of nucleotide sequence.
**PBMC**	Peripheral blood mononuclear cell, comprises of blood cell with round nucleus including lymphocytes (B cells, T cells, NK cells) and monocytes.
**Plasmonic**	The interaction which emerges from the coupling of visible light and free electrons in metallic nanostructures. The plasmonic effect generates a localized heating for precise cell membrane permeabilization.
**Shear force**	A force in a parallel direction to the contact surface. Fluid flowing around the cells generates this type of force which may permeabilize the cell membrane.
**Stable transfection**	A result of transfection in which the gene is integrated into the cells genome, thus the modification will be passed down to daughter cells. Gene therapy mainly aims for stable transfection.
**Surface acoustic wave**	An acoustic wave propagating on the surface of the materials. SAW is preferable for microfluidics technology due to its controllability and ease of integration in microsystem environment.
**Suspension cell**	A type of cell which grows free-floating in the suspension medium, such as hematopoietic cell lines. Suspension cells are traditionally considered hard to transfect.
**Transfection**	A process to introduce nucleic acids (DNA or RNA) and proteins into mammalian cells in order to change the cells' behavior.
**Transient transfection**	A result of transfection in which the gene is not integrated into the genome, thus modification is transient for a limited period of time.

**Table 2:** CRISPR/Cas9 Delivery Formats

Format	Time to Cas9 Onset Expression	Advantages	Disadvantages
pDNA	5 hours	Low cost of production Prolonged expression of CRISPR components	High possibility of insertional mutagenesis High risk of off-target effects
mRNA	1 hour	No insertional mutagenesis Fast expression of CRISPR components	High cost of production
RNP	Immediately	High editing efficiency Low risk of off-target effects	High cost of production

**Table 3:** Major Viral Vectors for Gene Delivery

Viral vectors	Genome Type and Capacity	Advantages	Disadvantages
Adenovirus	dsDNA8 kb	Effective transduction to non-dividing cells	High immunogenicity No genome integration into host cell
Adeno-associated virus	ssDNA4 kb	Effective transduction to non-dividing cells Low immunogenicity Ability to transduce diverse cell types Nonpathogenic	Small packaging capacity, may require co-transduction for CRISPR
Retrovirus	ssRNA8 kb	Genome integration into host cell Low immunogenicity Ability to transduce diverse cell types	Unsuitable to non-dividing cells High possibility of insertional mutagenesis
Lentivirus	ssRNA8 kb	Effective transduction to non-dividing cells Genome integration into host cell	High possibility of insertional mutagenesis

**Table 4:** Ongoing and Completed Clinical Trials Employing Physical Methods for Gene Delivery

ClinicalTrials.gov Identifier	Physical Methods	Cargo Type	Target Cells	Start Year	Aim
NCT00684294	Electroporation	plasmid DNA	Tumor cells	2008	Tumor cell vaccine for advanced cancer
NCT01530698	Electroporation	mRNA	Dendritic cells	2010	Dendritic cell vaccine for melanoma
NCT00968760	Electroporation	plasmid DNA	T cells	2011	T cell immunotherapy for B lymphoma
NCT01456104	Electroporation	mRNA	Dendritic cells	2011	Dendritic cell vaccine for melanoma
NCT02315118	Electroporation	mRNA	T cells	2014	T cell immunotherapy for B lymphoma
NCT01995708	Electroporation	mRNA	Dendritic cells	2014	Dendritic cell vaccine for multiple myeloma
NCT02117518	Electroporation	mRNA	T cells	2014	Immunotargeting of reactive T cells which induces Type 1 Diabetes
NCT03083054	Electroporation	mRNA	Dendritic cells	2016	Dendritic cell vaccine to suppress leukemia progression
NCT03166878	Electroporation	mRNA	T cells	2017	Universal CAR-T cell for B lymphoma
NCT03415100	Electroporation	mRNA	Natural killer cells	2018	Enhancing cell therapy specificity and activity for metastatic solid tumors
NCT03399448	Electroporation	RNP	T cells	2018	Enhancing immune activity of the T cells toward refractory cancer
NCT04084951	Mechanical	Protein	PBMC	2020	Enhancing immune response against tumor cells expressing HPV16 E6 and E7

**Table 5:** Representative Commercial Products Utilizing Physical Transfection

Physical Methods	Company	Products	Product Type	Technology
Mechanical	Indee Labs	*Under development*	Device	Microfluidic vortex shedding
Mechanical	CellFE	*Under development*	Device	Microfluidic constriction
Mechanical	SQZ Biotech	SQZ-PBMC-HPV	Cell therapy	Microfluidic constriction
Mechanical & Electrical	OpenCell Technologies	POROS-EP	Device	Combination of acoustic shear poration and electrophoresis
Electrical	Lonza	Nucleofector	Device	Conventional electroporation
Electrical	Biorad	Gene Pulsar	Device	Conventional electroporation
Electrical	ThermoFisher Scientific	Neon Transfection System	Device	Conventional electroporation
Electrical	Miltenyi Biotec	CliniMACS® Electroporator	Device	Automated conventional electroporation
Electrical	MaxCyte	MaxCyte STX®	Device	Flow electroporation
		CARMA™	Cell therapy	
Electrical	Celetrix	CTX Electroporator	Device	Tube electroporation
Electrical	Infinitesimal	NFP-E™	Device	Nanostructure-mediated electroporation
Electrical	NAVAN Technologies	*Under development*	Device	Nanostructure-mediated electroporation
Laser/Thermal	Cellino Biotech	*Under development*	Device	Nanostructure-mediated plasmonic poration
Magnetic	OZ Biosciences	Magnetofection™	Reagent	Magnetic particle-assisted transfection
Magnetic	PromoCell	MATra	Reagent	Magnetic particle-assisted transfection

**Table 6:** Comparison of Current Physical Transfection Methods for CRISPR/Cas9

Physical Methods	Advantages	Drawbacks	Suitable cargo	Recommended Cell Condition
Mechanical (non-microinjection)	Effective to difficult-to-transfect cells Simple setup and infrastructure Do not require external field supply	Ineffective delivery for large molecules	Protein mRNA	Suspension
Electrical	Effective to difficult-to-transfect cells Applicable for *in situ* transfection	May damage cells and cargo molecules	pDNA mRNA	Suspension (bulk) Adherent (micro/nano)
Acoustoporation	High cell viability	Limited throughput Low efficiency	Protein mRNA	Adherent
Laser / optothermal	High spatial control	Low throughput May damage cells at certain wavelengths	Protein mRNA	Adherent
Magnetic	Effective to difficult-to-transfect cells Applicable for *in situ* transfection	Require chemical complex formation	pDNA mRNA	Adherent

**Table 7:** Summary of Physical Transfection Techniques for CRISPR Delivery

Transfection Method	Cell Type	CRISPR Format & Dosage	Gene Editing Performance	Target Gene	Ref.
Mechanical	SU-DHL-1 lymphoma cells	Plasmid , @ -	70% knockout of fluorescence expression, assessed via flow cytometry	EGFP	88
	MDA-MB-231 basal cells	Plasmid , @ -	90% knockout of fluorescence expression, assessed via flow cytometry	EGFP	
	SK‐BR‐3 cells	RNP, @ 2 µM	80% knockout of fluorescence expression, assessed via flow cytometry	EGFP	90
	MDA-MB-231 basal cells	RNP, @ 2 µM	43% editing, assessed by surveyor mutation detection assay	pMAPKs	
	SUM-159 cells	RNP, @ 2 µM	47% editing, assessed by surveyor mutation detection assay	pMAPKs	
	Human primary T cells	RNP, @ 2 µM	36% editing, assessed by surveyor mutation detection assay	pMAPKs	
			~35% knockout of PD-1 surface expression, assessed via flow cytometry	PD-1	
	Human HSCs	RNP, @ 2 µM	-	C/EBPα	87
	Human HSCs	RNP, @ 25 µM	63% knockout of β2-microglobulin surface expression, assessed via flow cytometry	B2M	89
	Human primary T cells	RNP, @ 0.625 µM	55% editing, assessed by T7E1 assay	PD1	91
Electrical	HEK 293 cells	Plasmid, @ 200 µg/mL	-	-	92
	Human primary CD4^+^ T cells	RNP @ ~ 50 µM	96% knockout of CXCR4 surface expression, assessed via flow cytometry	CXCR4	110
			98% knockout of CD127 surface expression, assessed via flow cytometry	CD127	
			94% knockout of CCR7 surface expression, assessed via flow cytometry	CCR7	
	Human primary CD8^+^ T cells		95% knockout of PD1 surface expression, assessed via flow cytometry	PD1	
			96% knockout of TIGIT surface expression, assessed via flow cytometry	TIGIT	
			98% knockout of CTLA4 surface expression, assessed via flow cytometry	CTLA4	
	Mouse CD4^+^ T cells		90% knockout of CD90 surface expression, assessed via flow cytometry	CD90	
			88% knockout of CTLA4 surface expression, assessed via flow cytometry	CTLA4	
	Mouse CD8^+^ T cells		98% knockout of CD8α surface expression, assessed via flow cytometry	CD8α	
			93% knockout of CTLA4 surface expression, assessed via flow cytometry	CTLA4	
	Human iPSCs	RNP, @ 0.5 µM	52% editing of APP, assessed by T7E1 assay	APP	94
			70% editing of AAVS1, assessed by T7E1 assay	AAVS1	
			59% editing of OCT4, assessed by T7E1 assay	OCT4	
			52% editing of PD1, assessed by T7E1 assay	PD1	
			56% editing of APP, assessed by DNA sequencing90% editing of APP with HDR DNA template, assessed by DNA sequencing48% editing of PD1, assessed by DNA sequencing62% editing of PD1 with HDR DNA template. assessed by DNA sequencing		
	Human MSCs	RNP, @ 0.5 µM	45% editing of B2M, assessed by T7E1 assay30% knockout of β2-microglobulin surface expression, assessed via flow cytometry77% knockout of β2-microglobulin surface expression with HDR DNA template, assessed via flow cytometry	B2M	
	Human primary T cells	RNP, @ 0.5 µM	57% editing of PD1, assessed by T7E1 assay14% knockout of PD1 surface expression, assessed via flow cytometry19% knockout of PD1 surface expression with HDR DNA template, assessed via flow cytometry	PD-1	
	HEK 293 cells	RNP, @ 10 µM	31% editing, assessed by T7E1 assay33% editing, assessed by Sanger sequencing	PPIB	95
	HeLa cells	RNP, @ 10 µM	24% editing, assessed by T7E1 assay	PPIB	101
	Jurkat cells	RNP, @ 10 µM	26% editing, assessed by T7E1 assay		
Acoustic	HEC-1A cells	Plasmid, @ 2 μg/mL	57% knockout of mRNA expression, assessed via western blot	C-erbB-2	118
	HeLa & HEK 293 cells	Plasmid, @ 110 ng/μl	-	-	121
Magnetic	Porcine fibroblast	Plasmid, @ 375 ng/mL & 750 ng/mL	15% & 27% editing, assessed by Sanger sequencing	H11 locus	127
	Hepa 1-6 cell	Plasmid in baculovirus	35% editing, assessed by T7E1 assay	Vegfr2	129
Laser/ Plasmonic	SC1 cells	sgRNA (to Cas9 expressing cells), @ 5 µM	12% editing, assessed by T7E1 assay30% knockout of CCR7 surface expression, assessed via flow cytometry	CCR7	141
		RNP, @ 5 µM	6% editing, assessed by T7E1 assay22% knockout of CCR7 surface expression, assessed via flow cytometry		
	Mouse primary T cells	RNP, @ 5 µM	4% knockout of CXCR3 surface expression, assessed via flow cytometry	CXCR3	
	Lymph node stroma cells	RNP, @ 5 µM	5% knockout of fluorescence expression, assessed via flow cytometry	EGFP	
